# Role of RGO support and irradiation source on the photocatalytic activity of CdS–ZnO semiconductor nanostructures

**DOI:** 10.3762/bjnano.7.161

**Published:** 2016-11-11

**Authors:** Suneel Kumar, Rahul Sharma, Vipul Sharma, Gurunarayanan Harith, Vaidyanathan Sivakumar, Venkata Krishnan

**Affiliations:** 1 School of Basic Sciences and Advanced Materials Research Center, Indian Institute of Technology Mandi, Kamand, Mandi 175005, H.P., India; 2 Department of Chemistry, National Institute of Technology, Rourkela, Odisha, India

**Keywords:** catalytic properties, chemical synthesis, nanostructures, semiconductors, transmission electron microscopy (TEM)

## Abstract

Photocatalytic activity of semiconductor nanostructures is gaining much importance in recent years in both energy and environmental applications. However, several parameters play a crucial role in enhancing or suppressing the photocatalytic activity through, for example, modifying the band gap energy positions, influencing the generation and transport of charge carriers and altering the recombination rate. In this regard, physical parameters such as the support material and the irradiation source can also have significant effect on the activity of the photocatalysts. In this work, we have investigated the role of reduced graphene oxide (RGO) support and the irradiation source on mixed metal chalcogenide semiconductor (CdS–ZnO) nanostructures. The photocatalyst material was synthesized using a facile hydrothermal method and thoroughly characterized using different spectroscopic and microscopic techniques. The photocatalytic activity was evaluated by studying the degradation of a model dye (methyl orange, MO) under visible light (only) irradiation and under natural sunlight. The results reveal that the RGO-supported CdS–ZnO photocatalyst performs considerably better than the unsupported CdS–ZnO nanostructures. In addition, both the catalysts perform significantly better under natural sunlight than under visible light (only) irradiation. In essence, this work paves way for tailoring the photocatalytic activity of semiconductor nanostructures.

## Introduction

In the past decade, there has been an increased interest in the photocatalytic degradation of various kinds of organic pollutants in water and soil [1]. Many of these pollutants, particularly dyes, are carcinogenic and mutagenic [2]. Thus, there is an urgent need for removal of these pollutants as these are harmful to both human and environment [3]. Previous studies have extensively explored the role of semiconductor oxides mainly ZnO, TiO_2_ in the photocatalytic decomposition of organic pollutants [4–8]. These semiconductor photocatalysts not only degrade the contaminants, but also cause their complete mineralization into CO_2_, H_2_O and mineral acids [9–10]. Thus, it is advantageous over physico-chemical methods such as flocculation–coagulation [11], ozonization [12] and adsorption [13], as these methods are unable to remove the contaminants completely. Some recent studies have reported ZnO as a better photocatalytic material in the degradation of organic dyes in aqueous solutions, because of high charge carrier mobility and significantly longer electron life time than TiO_2_ [14–16].

Zinc oxide is a well-known semiconductor with a band gap energy of 3.37 eV and has been widely explored as photocatalytic material due to its non-toxic nature, high exciton binding energy (60 meV), photosensitivity and stability on exposure to high energy radiation [17]. Due to this high band gap value, ZnO can only absorb ultraviolet (UV) light and this limits its practical applications [18]. Thus, in order to design more efficient photocatalysts, which are active in visible light, many research groups have devoted their studies towards dye sensitization [19], ion doping [20] and coupling of semiconductors [21]. Recently, coupling of the semiconductors have attracted much attention and it has been proved that this coupling efficiently increases the photocatalytic performance by reducing the recombination probability of photo-generated charge carriers, increasing the photo response range and enhancing the interfacial charge transfer [22].

We have focused our study on the coupling of ZnO nanorods with nanoparticles of CdS, a semiconductor active under visible light, to form coupled CdS–ZnO heterojunction nanostructures. The CdS nanoparticles are an attractive photocatalytic material for visible-light harvesting due to the narrow band gap (2.42 eV) [23]. But fast recombination of photo generated charge carriers and their aggregation to form large particles, limits the photocatalytic activity of CdS nanostructures [24]. Once these structures are coupled with other semiconductor materials to form nanocomposites, they turn out be an efficient photocatalyst [25]. Recently, there have been few reports available in literature on CdS–ZnO coupled photocatalytic systems with enhanced activity [26]. On illumination of light, charge transfer takes place from the conduction band (CB) of CdS to that of ZnO [22,27].

The CdS–ZnO semiconductor nanostructures can be further supported on graphene/reduced graphene oxide (RGO) materials to improve their photocatalytic properties. Ideally, graphene is a single layer carbon sheet, which consists of a two dimensional (2D) network of sp^2^-hybridized carbon atoms with hexagonal packed lattice structure [28]. Graphene also possesses unique electronic, optical and mechanical properties such as high theoretical specific surface area (2630 m^2^·g^−1^) [29], chemical stability, high transparency and good thermal conductivity (5000 W·m^−1^·K^−1^) [30]. Its optical transmittance is about 97.7% and possesses superior electron mobility (200000 cm^2^·V^−1^·s^−1^), which makes it an ideal material for photocatalyst support [31]. Several semiconductor nanocomposites supported on graphene have been used as photocatalysts for the degradation of organic pollutants [32–35]. In one of our recent works [34], we have reported the synergistic effect of MoS_2_–RGO support to improve the photocatalytic performance of ZnO nanoparticles. However, the role played by RGO support in enhancing the photocatalytic performance of the nanocomposites has not been fully explored. Furthermore, the photocatalytic activity is also influenced by the irradiation source. With regard to this, we focused our studies on determining the role of the RGO support and the irradiation source on the photocatalytic activity of CdS–ZnO semiconductor nanostructures. Another unique aspect of this work is the formation of efficient binary and ternary heterojunctions having nanoparticles (NP), nanorods (NR) and nanosheets (NS), comprising of CdS, ZnO and RGO, respectively. In this work, , the preparation and detailed characterization of binary and ternary nanocomposites are presented and their photocatalytic activity have been demonstrated with respect to the degradation of methyl orange (MO) dye, both under visible light (only) irradiation from a solar simulator and natural sun light. The obtained results have been discussed in detail, and the role of RGO support and irradiation source on the photocatalytic activity of CdS–ZnO nanostructures has been elucidated.

## Experimental

### Materials

For the synthesis of GO graphite powder (crystalline, −300 mesh, 99%) was purchased from Alfa Aesar, whereas sodium nitrate (NaNO_3_), sulfuric acid (H_2_SO_4_), potassium permanganate (KMnO_4_) and hydrogen peroxide (H_2_O_2_) were purchased from Merck. Zinc chloride (ZnCl_2_), sodium hydroxide (NaOH), cadmium acetate dihydrate (Cd(OOCCH_3_)_2_·2H_2_O), sodium sulfide (Na_2_S), ammonia solution and methyl orange were also supplied by Merck. Polyvinyl pyrrolidone (PVP) used in synthesis was purchased from Sigma-Aldrich. All chemicals were used as received without further purification. Deionized water (18.2 MΩ·cm) used in synthesis was obtained from a double-stage water purifier (ELGA PURELAB Option-R7).

#### Synthesis of graphene oxide

Graphene oxide (GO) was synthesized from natural graphite flakes using a modified Hummers’ method [36]. As described in literature [34], 1.0 g of graphite powder and 0.5 g of NaNO_3_ was stirred in 23 mL of concentrated H_2_SO_4_ in an ice bath to maintain a reaction temperature below 10 °C. This was followed by the slow addition of 3.0 g of KMnO_4_ to the reaction mixture with continuous stirring. Subsequently, the reaction mixture was stirred in an oil bath at 35 °C until a brown colored paste was formed after about 4 h. The reaction was terminated by slow addition of deionized water (90 mL), which increased the temperature to 95–98 °C and resulting suspension was maintained at this temperature for 15–20 min; subsequently, the suspension was then diluted to about 250 mL by the addition of deionized water. This is followed by the addition of 10 mL H_2_O_2_ to remove unreacted KMnO_4_ in the reaction mixture. In order to remove the ions of oxidant origin, the mixture was washed with 10% HCl and then with deionized water until pH value of the filtrate was neutral. Obtained graphite oxide was subjected to ultrasonication for its exfoliation followed by centrifugation at 4500 rpm for 15 min. The final product was obtained by drying with rotary evaporator at 40 °C followed by vacuum drying overnight at same temperature.

#### Synthesis of ZnO nanorods

ZnO nanorods (NR) were synthesized through a previously reported solvothermal method [37]. In brief, 10 mL of 0.2 M zinc chloride (ZnCl_2_) solution in ethanol was added into 70 mL of 0.5 M sodium hydroxide (NaOH) solution dropwise under vigorous stirring. This was followed by ultrasonic treatment of the solution for 30 min for homogenization. Then, this homogenous solution was transferred to a 100 mL teflon-lined stainless steel autoclave, sealed tightly and maintained at 180 °C for 12 h. White precipitates of ZnO NR were collected by centrifugation and washed several times with deionized water and ethanol and finally dried at 60 °C.

#### Synthesis of CdS nanoparticles

CdS nanoparticles were synthesized as per a previously reported method [38]. In a typical procedure, about 20 mL of cadmium acetate dihydrate (Cd(OOCCH_3_)_2_·2H_2_O) (0.2 M) was prepared in deionized water. To this solution, 20 mL of sodium sulfide (Na_2_S) solution (0.2 M) was added dropwise under continuous stirring. After 10 min of stirring, 0.5 g of PVP was added as capping agent under vigorous stirring. The pH value of the solution was maintained at around 10 by adding ammonia solution. The resultant solution was refluxed for about 1 h at 70 °C. Upon completion of the reaction, the product was washed with deionized water and ethanol thrice and finally yellow colored CdS NP were formed after drying in an oven at 80 °C for 2 h.

#### Synthesis of CdS–ZnO binary nanocomposite

CdS–ZnO binary nanocomposite was prepared by employing a reported hydrothermal strategy [39]. In short, 0.2 M ZnCl_2_ was dispersed in 40 mL deionized water, followed by the addition of 0.5 M NaOH solution dropwise with continuous stirring. Aqueous ammonia was added to maintain the pH value around 8. Finally, Cd(OOCCH_3_)_2_·2H_2_O (0.2 M) and 4 mL of thioglycolic acid (0.2 M) was added into 40 mL of above solution under vigorous stirring. Subsequently, this homogenous suspension was transferred to 50 mL teflon-lined stainless steel autoclave and kept at 140 °C for 48 h. After the completion of reaction time, the product was collected by centrifugation and washed with ethanol and deionized water thrice, and dried in vacuum. Based on the molar ratio of the precursors used in the synthesis, the ratio of CdS to ZnO is expected to be 1:1 in this binary nanocomposite.

#### Synthesis of CdS–ZnO–RGO ternary nanocomposite

For the synthesis of the CdS–ZnO–RGO ternary nanocomposite, about 0.01 g of as prepared GO was dispersed in ethanol by ultrasonication. Then, 0.2 g of previously prepared CdS–ZnO was added to the GO solution under vigorous stirring for 2 h to obtain homogenous suspension. Once homogenization is achieved, the suspension is transferred to teflon-lined stainless steel autoclave and kept at 120 °C for 24 h. The desired product was obtained after washing with water and dried at 60 °C. Based on the molar ratio of the precursors used in the synthesis, the ratio of CdS to ZnO is expected to be 1:1 while having 1 wt % of RGO in this ternary nanocomposite.

### Photocatalytic activity

Photocatalytic activities of as prepared photocatalysts were evaluated by monitoring the decomposition of MO dye at room temperature. Typically, 10 mg of photocatalysts (CdS–ZnO or CdS–ZnO–GO) was added to 50 mL of the aqueous solution of MO (10^−5^ M). Initially, the suspension was magnetically stirred in the dark for 30 min to attain adsorption–desorption equilibrium. Subsequently, the suspension was continuously stirred under visible light irradiation. For one set of experiments, a solar simulator (OAI Trisol, AM 1.5, 100 mW·cm^−2^) having a UV cut-off filter (λ > 420 nm) was used for visible light illumination. Another set of experiments were performed under natural sunlight and the light intensity was measured using a LX-101A digital luxmeter. At periodic time intervals, the photo-reacted suspension (ca. 1 mL) was analyzed by recording the absorbance using a UV–vis spectrophotometer.

### Characterizations

The UV–visible absorption spectra of the samples were recorded using Shimadzu UV-2450 spectrophotometer in the wavelength range from 200 to 800 nm. Fourier transform infrared (FTIR) spectra were collected using Agilent K8002AA Carry 660 FTIR instrument. Optical properties were analyzed by UV–vis diffuse reflectance spectroscopy (DRS) using a Perkin Elmer UV/VIS/NIR Lambda 750 spectrophotometer, in which polytetrafluroethylene (PTFE) polymer was employed as internal reflectance standard. Morphology of the samples was characterized using a field-emission scanning electron microscope (FESEM) JFEI Nova Nano SEM-450 and a high resolution transmission electron microscope (HRTEM) FEI Tecnai G2 20 S-twin microscope operating at 200 kV. Energy dispersive X-ray spectroscopy (EDAX) was obtained using the same HRTEM instrument. X-ray diffraction (XRD) measurements were done using the Agilent Supernova X-ray diffractometer using Ni-filtered Cu Kα irradiation (λ = 0.1542 nm) at 45 kV and 40 mA in 2θ ranging from 5 to 80° with a scan rate of 2°·min^−1^.

## Results and Discussion

### Powder X-ray diffraction analysis

**Figure 1:**
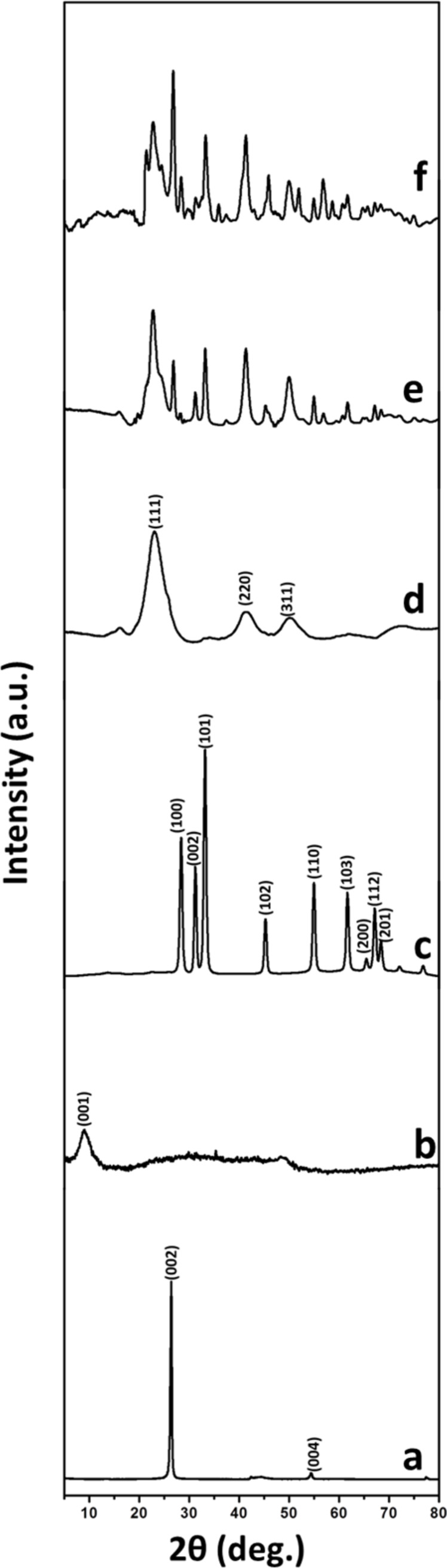
XRD patterns of (a) graphite powder, (b) GO, (c) ZnO NR, (d) CdS NP, (e) CdS–ZnO and (f) CdS–ZnO–RGO nanocomposite.

In order to investigate the crystalline phase of as prepared nanocomposites, powder X-ray diffraction (XRD) analysis was performed. Figure 1 presents the XRD patterns of graphite, GO, ZnO NR, CdS NP, CdS–ZnO and CdS–ZnO–RGO nanocomposite. Graphite powder shows a very strong peak at 2θ = 26.5°, which could be assigned to (002) reflection plane corresponding to the interlayer distance of about 0.34 nm. In addition to this peak there is another (004) reflection peak at 2θ = 54.3° corresponding to the interlayer distance of 0.17 nm [40]. After the oxidation of graphite, the interlayer distance increases mainly due to the introduction of hydroxy, epoxy and carbonyl groups, which is indicated by the characteristic (001) reflection peak of about 2θ = 8.5°, which correspond to an interlayer distance of about 1.08 nm [41]. The disappearance of characteristic (002) reflection peak and appearance of (001) reflection peak confirms the oxidation of graphite and formation of GO with well-defined lamellar structure [42–43]. This interlayer distance weakens the van der Waal interactions between sheets and makes exfoliation possible [44]. Once GO is reduced to RGO during hydrothermal treatment, the (002) reflection peak of GO disappears. The XRD patterns of ZnO nanorods show peaks at 2θ = 31.67, 34.31, 36.14, 47.40, 56.52, 62.73, 66.28, 67.91 and 69.03°, which could be assigned to the (100), (002), (101), (102), (110), (103), (200), (112) and (201) lattice planes, respectively, indicating the prepared ZnO NR have polycrystalline wurtzite structure (JCPDS no. 36-1451) [37]. The powder XRD pattern of the prepared CdS NP shows three diffraction peaks at 2θ = 26.8, 44.1 and 55.2°, which corresponds to the (111), (220) and (311) planes of hexagonal CdS (JCPDS 42-1411) [23]. Furthermore, in the powder XRD data of nanocomposites, all peaks that are ascribable to ZnO and CdS structures are evident, which demonstrates that the same crystal phases are retained in both binary (CdS–ZnO) and ternary (CdS–ZnO–RGO) nanocomposites. In the ternary composite, the characteristic peak of GO around 2θ = 10° is absent confirming its reduction to RGO. The characteristic diffraction peak around 2θ = 24° due to RGO is also absent in the ternary composite, which indicates that RGO sheets are not stacked due to the CdS–ZnO composite which inhibits the stacking.

### UV–vis diffuse reflectance spectroscopy (DRS)

**Figure 2:**
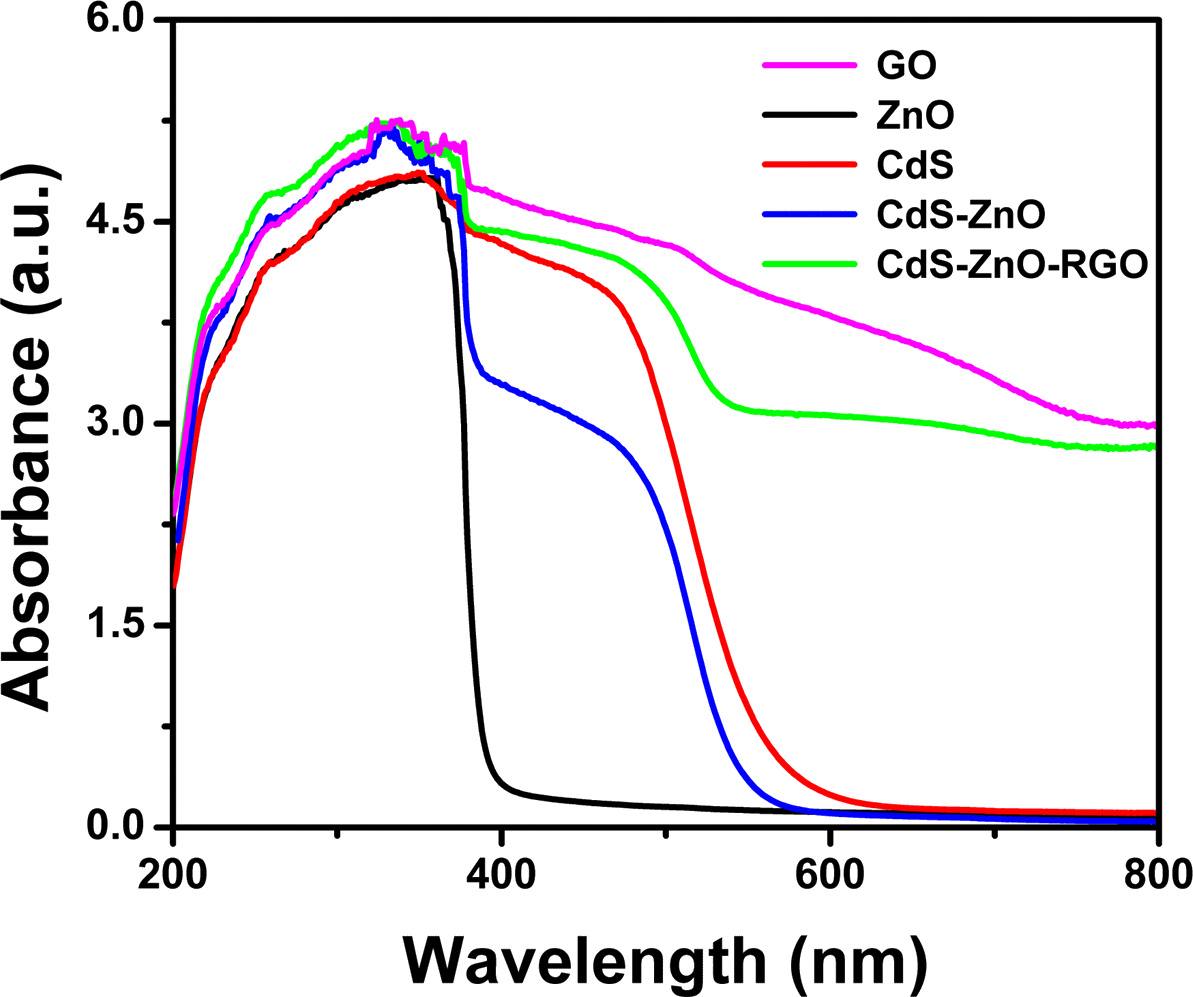
UV–vis diffuse reflectance spectra (DRS) of GO, ZnO NR, CdS NP, CdS–ZnO and CdS–ZnO–RGO nanocomposite.

**Figure 3:**
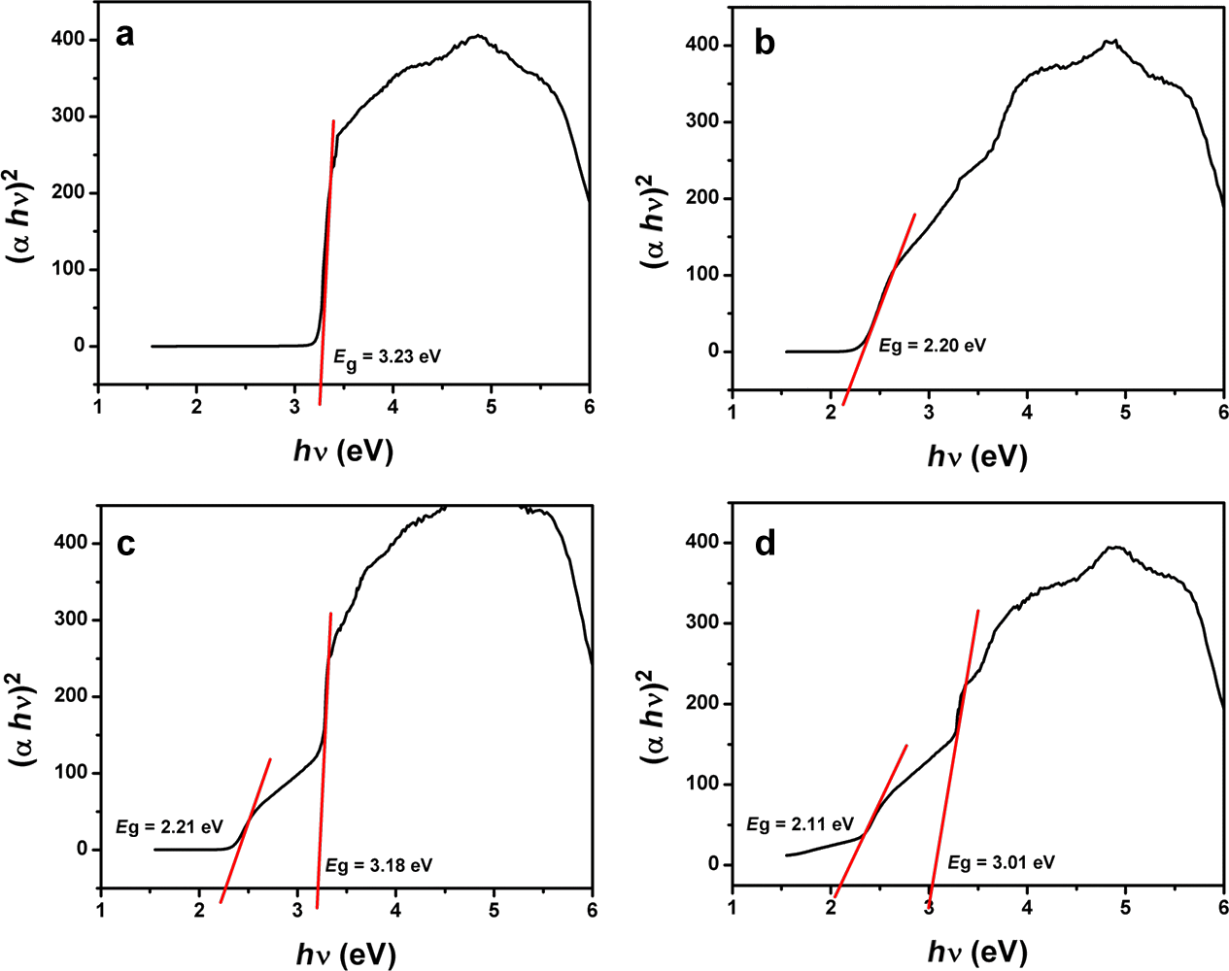
Plots of the transformed Kubelka–Munk function vs the energy of light: (a) ZnO NR, (b) CdS NP, (c) CdS–ZnO and (d) CdS–ZnO–RGO nanocomposite.

The optical properties of all prepared samples were analyzed by using UV–vis diffuse reflectance spectroscopy (DRS), the results of which are presented in Figure 2. It is clear from the DRS spectra that ZnO NR have an absorption band edge at 390 nm corresponding to a band gap value of 3.23 eV, which is in agreement with the reported band gap value of ZnO [45]. As-prepared CdS NP have an absorption edge around 580 nm, which corresponds to a band gap value of about 2.12 eV in agreement with the value reported for CdS [23]. Bare GO also shows the excellent light absorption in the range of 200–800 nm. Various studies have confirmed that the band gap of GO changes with the degree of oxidation [46–47]. The CdS–ZnO binary nanocomposite shows higher absorption in the visible light region compared to that of ZnO NR and exhibits an absorption edge in the range of 400–500 nm indicating the presence of CdS NP in the binary composite. One can observe a slight decrease in the band gap value of ZnO in this binary nanocomposite compared to its pristine form. The addition of GO to form the ternary composite (CdS–ZnO–RGO) results in continuous absorption in the region of 400–800 nm. Similar to the binary nanocomposite, in this case as well, two distinct absorption edges, corresponding to band gap values of 3.01 and 2.11 eV attributable to ZnO NR and CdS NP, respectively, could be evidenced. The enhanced absorption in visible light region can be attributed to chemical bonding between semiconductors and specific sites of carbon in GO resulting in charge delocalization and hence narrowing of the band gap of semiconductors [48–49]. The DRS spectra of ternary nanocomposite shows a broad elevated background in visible region, which is mainly due to GO because CdS does not show any absorption edge above its fundamental band edge (580 nm) [23]. Thus the presence of GO affects the optical properties of the ternary nanocomposite and is responsible for the red shift in the absorption spectrum, which ultimately results in narrowing of the band gap, which not only enhances light absorption in the visible light region, but also facilities efficient mobility of the charge carriers between the two semiconductors. Plots obtained by the transformation of the Kubelka–Munk function vs the energy of light are presented in Figure 3, which clearly shows the band gap narrowing in both the semiconductors due to the addition of GO.

### SEM analysis

**Figure 4:**
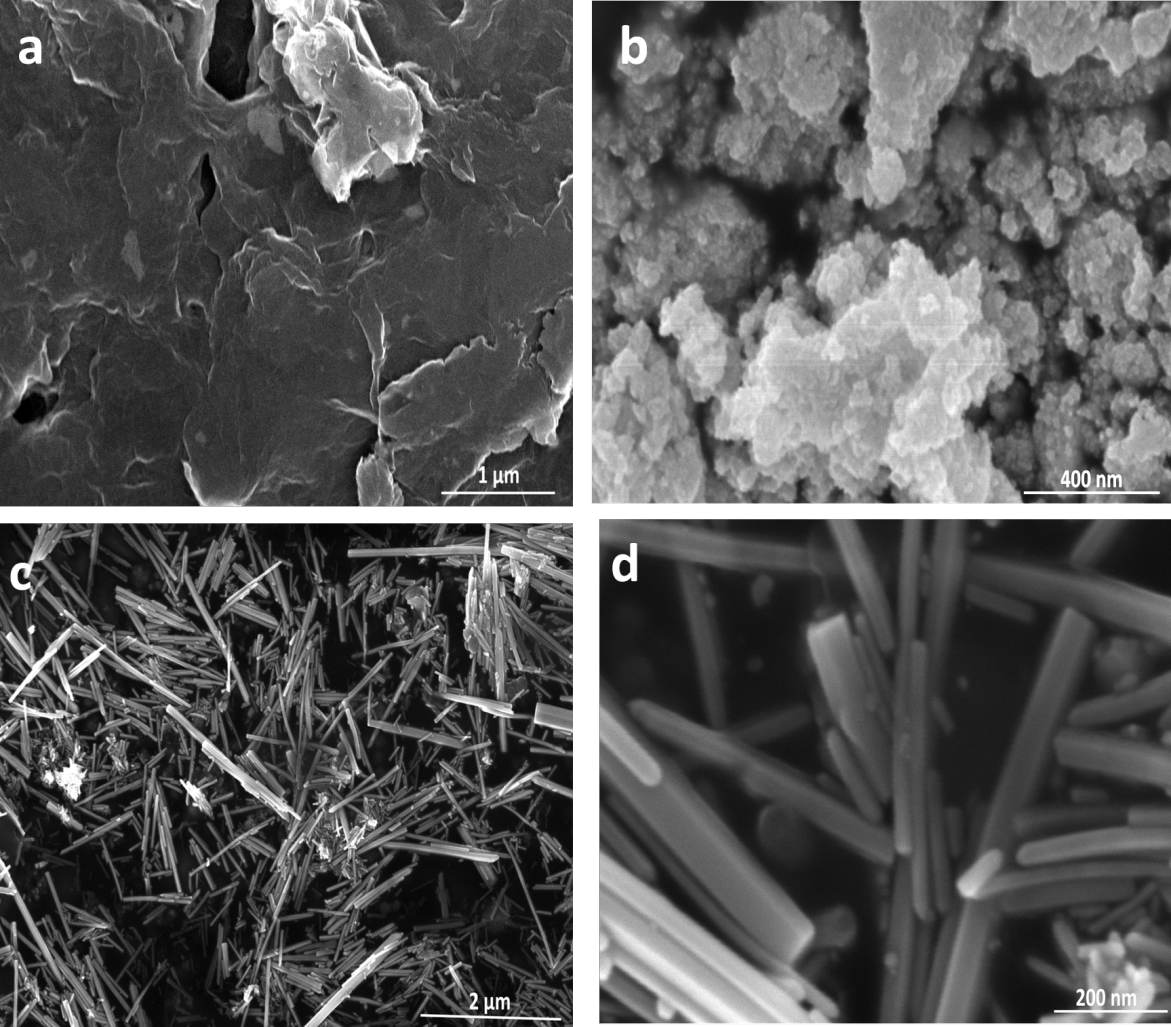
SEM images (a) GO sheet (b) CdS NP and (c, d) ZnO NR.

**Figure 5:**
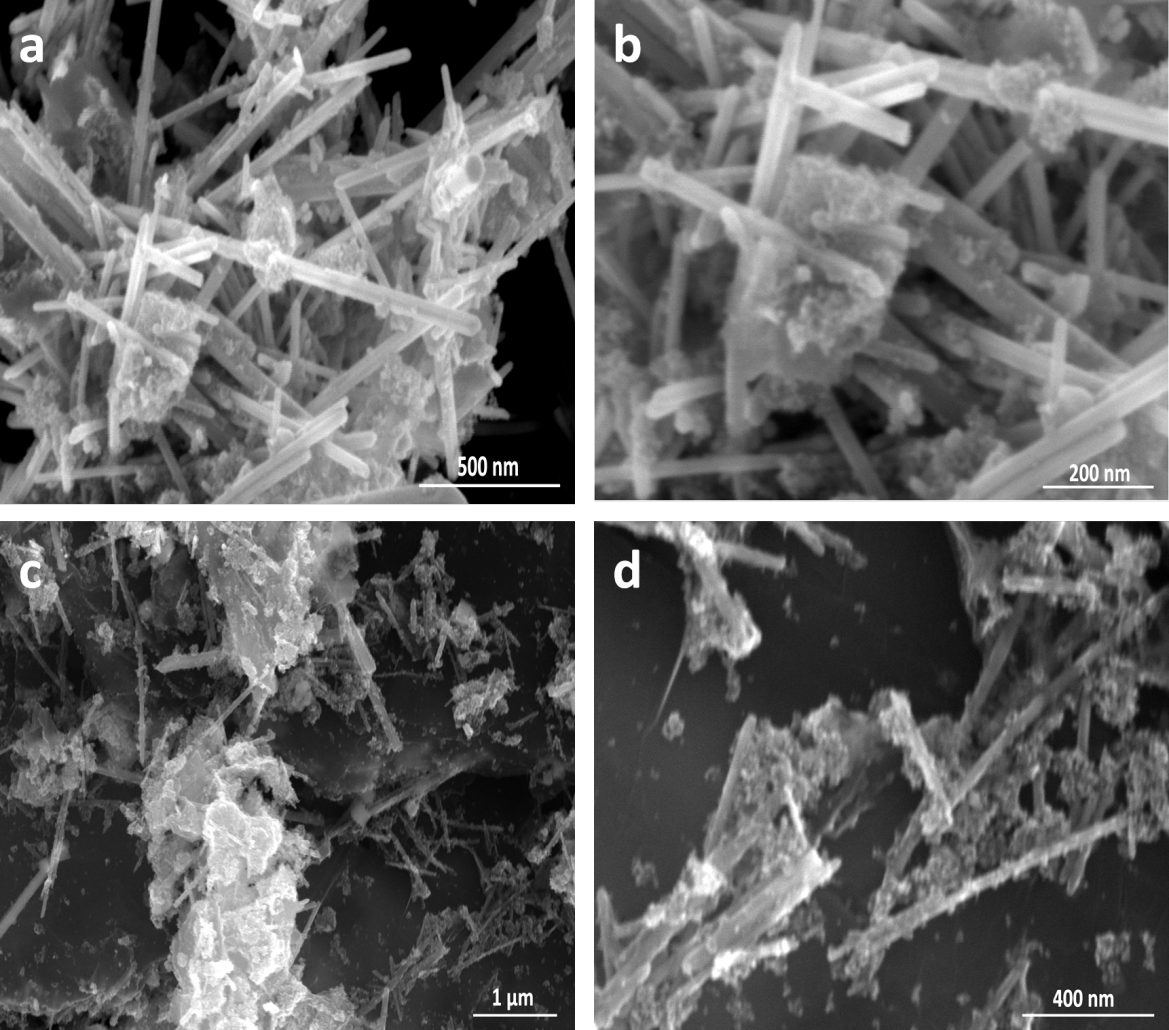
SEM images (a, b) CdS–ZnO binary nanocomposite and (c, d) CdS–ZnO–RGO ternary nanocomposite.

The surface morphology of all pristine materials as well as the binary (CdS–ZnO) and ternary (CdS–ZnO–RGO) nanocomposites was investigated by scanning electron microscopy (SEM). Figure 4a,b clearly shows the GO sheets and CdS NP, respectively. GO sheets show a flake-like morphology and the pristine CdS NP are agglomerated. ZnO has a rod-like morphology with lengths in the range of 2 to 3 µm and diameters from 30 to 50 nm (Figure 4c,d). In the preparation of nanocomposites, two semiconductors nanostructures (ZnO NR and CdS NP) are coupled first with each other to form a binary nanocomposite and then with GO sheets through hydrothermal method to form the ternary nanocomposite. Figure 5a,b indicate the coupling between CdS and ZnO, wherein CdS NP are present on the surface of ZnO NR. This binary nanocomposite (CdS–ZnO) on the surface of RGO can be seen in Figure 5c,d to form the ternary nanocomposite.

### TEM analysis

**Figure 6:**
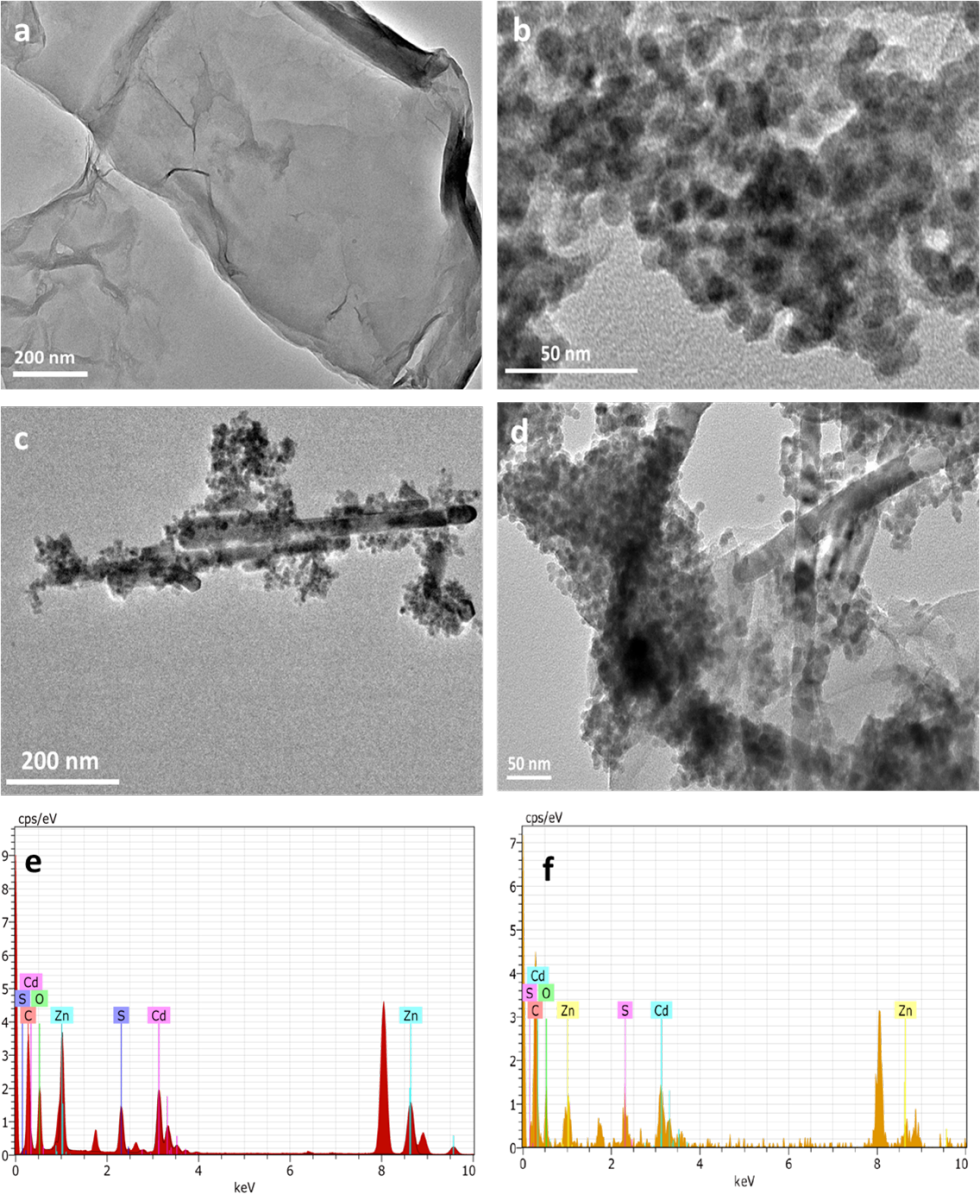
TEM Images (a) GO sheets, (b) CdS NP, (c) CdS–ZnO binary composite, (d) CdS–ZnO–RGO ternary composite and EDX spectrum (e) CdS–ZnO and (f) CdS–ZnO–RGO nanocomposites, respectively.

Transmission electron microscopy (TEM) analysis was performed to obtain further information on the surface morphology and microstructures of all prepared nanostructures (Figure 6). The TEM image of GO shows the flake-like shape (Figure 6a). The CdS NP are about 20 nm in size (Figure 6b). The TEM images of the CdS–ZnO binary and CdS–ZnO–RGO ternary nanocomposites are shown in Figure 6c and Figure 6d, respectively. It can be seen that the CdS–ZnO nanocomposite is distinctly coupled with RGO sheets and the original 2D structure of GO sheets is still retained even after hydrothermal treatment, which is in good agreement with literature reports [48]. The existence of all the constituent components in the final binary and ternary nanocomposites has been proved by the presence of corresponding peaks in the energy dispersive X-ray spectra (EDAX), as shown in Figure 6e and Figure 6f. The results obtained from TEM analysis corroborate well with both powder XRD and SEM characterizations.

### FTIR analysis

**Figure 7:**
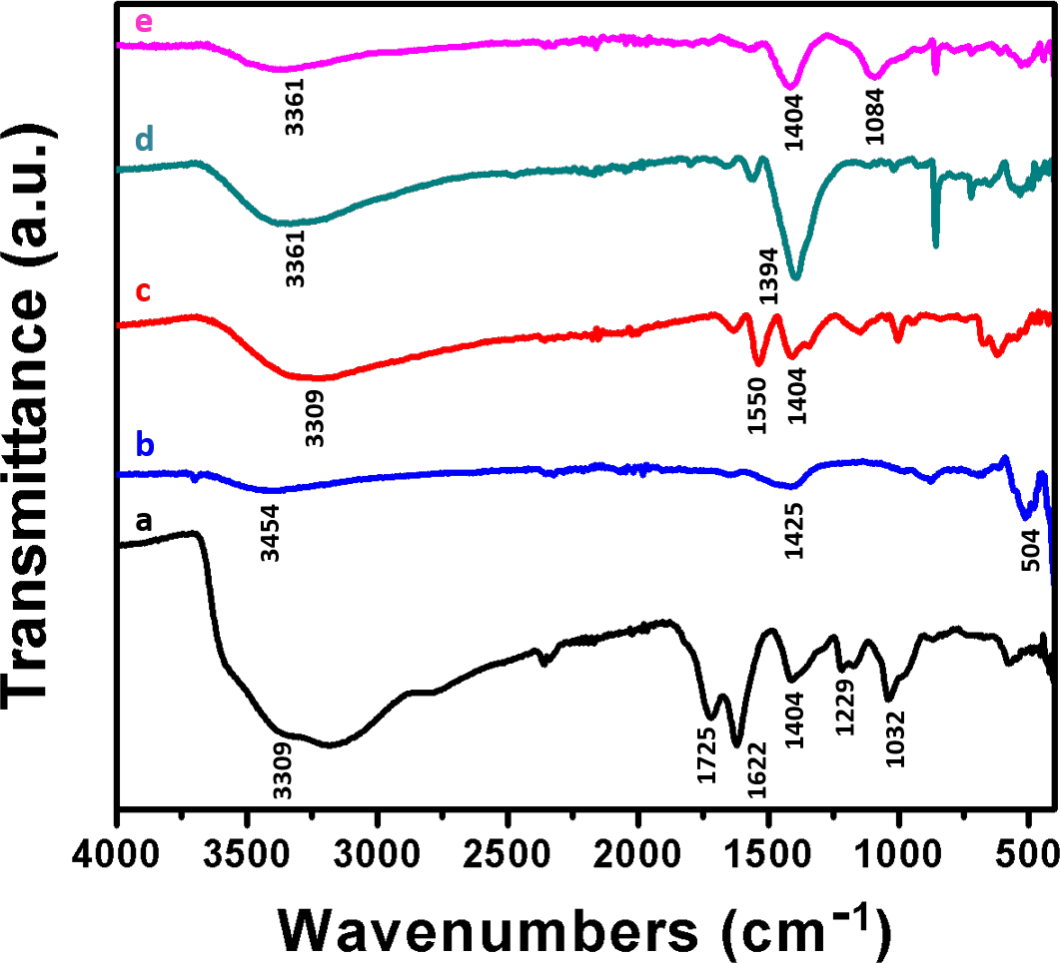
FTIR spectra of (a) GO, (b) ZnO, (c) CdS, (d) CdS–ZnO and (e) CdS–ZnO–RGO nanocomposite.

FTIR spectra for all samples are presented in Figure 7. The FTIR spectrum of GO shows strong broad absorption peaks at 3309 cm^−1^ and 1404 cm^−1^, which could be attributed to the O–H stretching vibrations and deformation vibrations of intercalated water [50]. Absorption peaks at 1032 cm^−1^, 1229 cm^−1^ and 1725 cm^−1^ are the characteristic stretching vibrations of C–O, epoxy C–O, and C=O of carbonyl groups, respectively, which are present at the edges of the GO sheets [51]. The absorption peak at 1622 cm^−1^ could be attributed to the aromatic C=C stretching vibrations in GO [51]. Similarly in FTIR spectra of ZnO NP, the broad absorption band at 3454 cm^−1^ and 1424 cm^−1^ could be attributed to O–H stretching and deformation of C–OH groups of water molecules. Zn–O bond stretching vibrations appears at 504 cm^−1^ [52]. FTIR spectra of CdS NP also reveal the presence of O–H stretching vibrations of adsorbed water molecules on its surface. The peak at 1550 cm^−1^ is attributed to the C–N stretching vibration of the PVP monomer, which was used as capping agent [53]. The 1404 cm^−1^ peak corresponds to the C–H bond of PVP [54]. CdS–ZnO nanocomposite also shows O–H stretching vibrations in the range of 3000–3500 cm^−1^ range and Zn–O stretching vibrations between 500 and 600 cm^−1^. The peak at 1394 cm^−1^ in the binary composite is assigned to C–H bonds of the capping agent. It is clear from the FTIR spectrum of CdS–ZnO–RGO nanocomposite that characteristic peaks of oxygen containing functional groups particularly at 1725 cm^−1^ are weakened and the O–H stretching peak decreases with some red shift. This is mainly attributed to the loss of oxygen containing functional groups and the reduction of GO to RGO after the hydrothermal treatment [55].

### UV–vis spectroscopic analysis

**Figure 8:**
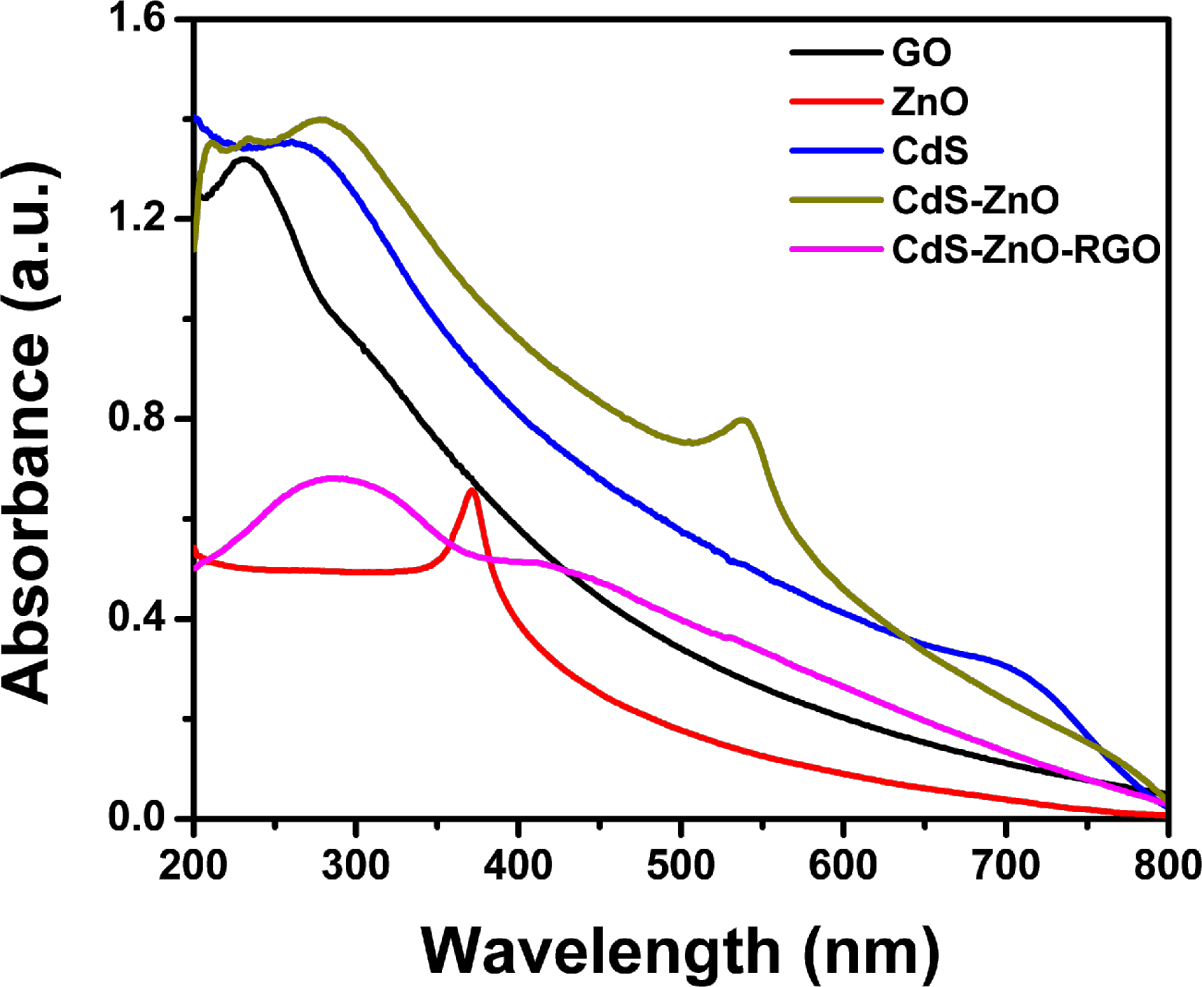
UV–vis absorption spectra of GO, ZnO NR, CdS NP, CdS–ZnO and CdS–ZnO–RGO nanocomposite.

Figure 8 shows the UV–vis spectra of GO, CdS, ZnO, CdS–ZnO and CdS–ZnO–RGO nanostructures. The UV–vis study of GO determined the degree of conjugation by λ_max_ value and shows two absorption peaks with maximum at 227–230 nm due to aromatic π→π* transition and a small shoulder at 303 nm due to the n→π* transition of carbonyl groups [56]. The absorbance peak of ZnO appears at 372 nm, which is in good agreement with literature reports [57]. UV–vis spectra for CdS NP shows a clear absorbance band in 400–500 nm range. which corresponds to the visible region of spectrum [58]. The UV–vis spectrum of the binary nanocomposite (CdS–ZnO) shows the absorption both in the UV and the visible region confirming the presence of both CdS and ZnO in the composite. Finally the absorption spectra of ternary nanocomposite (CdS–ZnO–RGO) shows an absorption near 250 nm, which indicates the red shift of the band at 227 nm in GO. This red shift is mainly due to the reduction of GO to RGO during the hydrothermal reaction, indicating an increase of the electronic conjugation [59]. Enhanced absorption of this ternary nanocomposite in visible region affirms the presence of all three components.

### Photocatalytic performance

**Figure 9:**
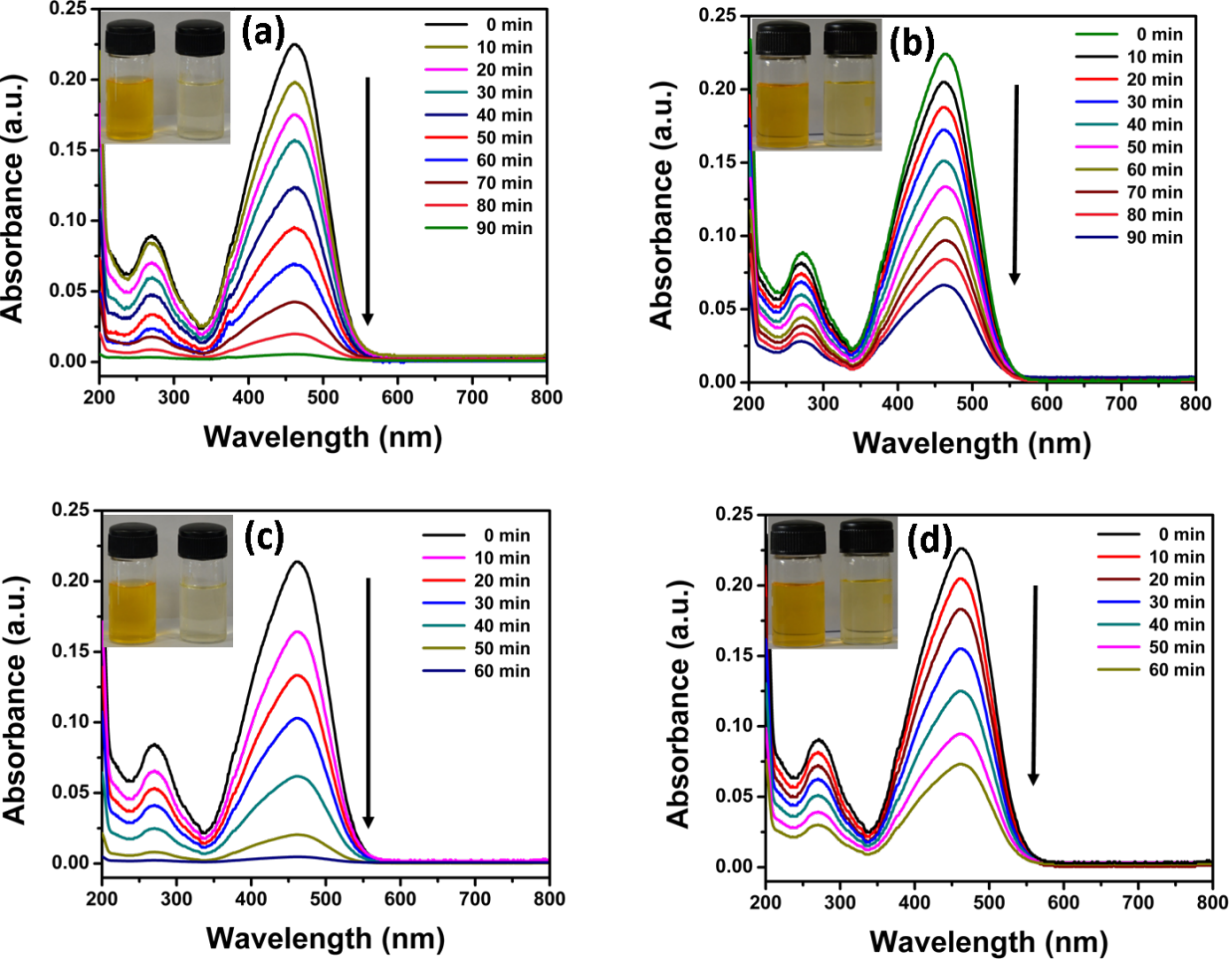
Time-dependent UV–vis spectra of photocatalytic degradation of MO: (a) visible light irradiation from a solar simulator using CdS–ZnO–RGO ternary nanocomposite, (b) visible light irradiation from a solar simulator using CdS–ZnO binary nanocomposite, (c) natural sun light irradiation using CdS–ZnO–RGO ternary nanocomposite and (d) natural sun light irradiation using CdS–ZnO binary nanocomposite. Experimental conditions: MO concentration 10^−5^ M, photocatalyst 10 mg per 50 mL.

**Figure 10:**
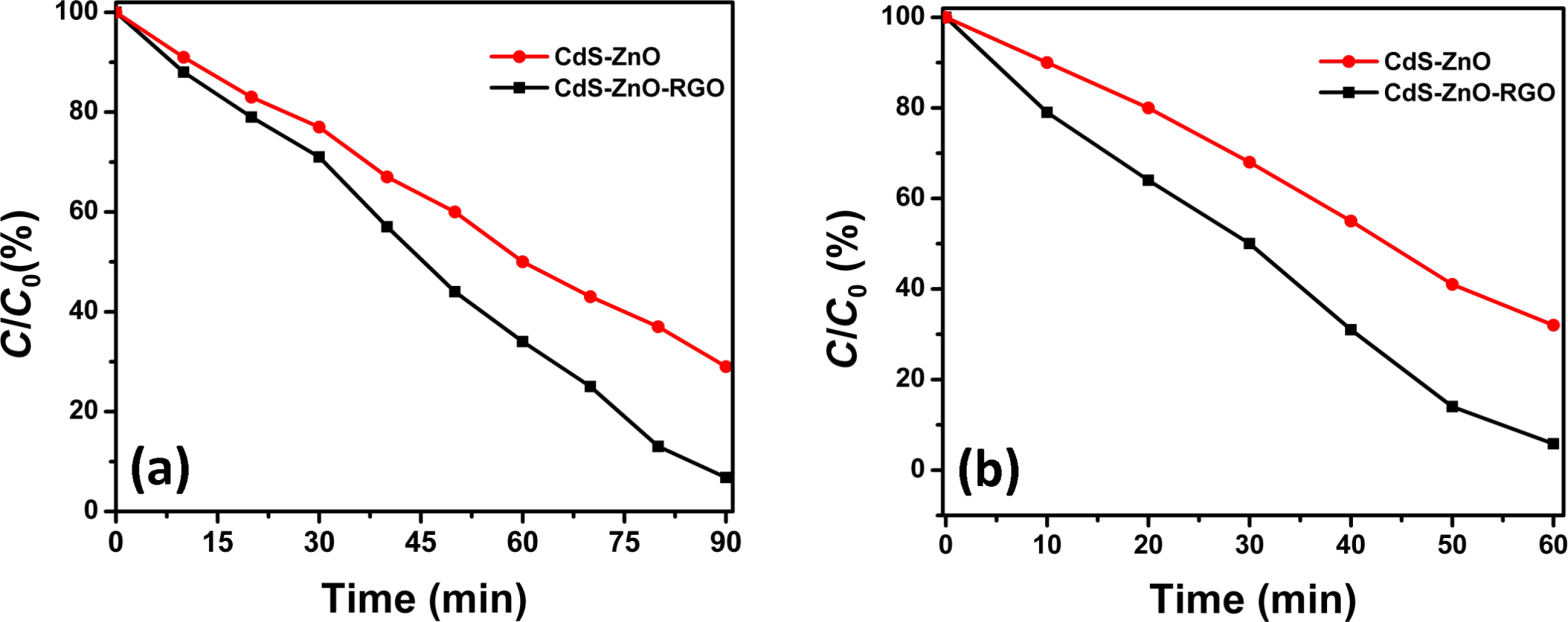
Kinetic curves for degradation of MO under (a) visible light irradiation from a solar simulator and (b) natural sunlight. The lines are to guide the eye.

The photocatalytic performance of CdS–ZnO binary and CdS–ZnO–RGO ternary nanocomposites is evaluated by measuring the photodegradation of methyl orange (MO), as model dye, under visible light irradiation from a solar simulator or under natural sun light. Prior to illumination, the suspension was equilibrated in dark for 30 min. During this period adsorption and desorption equilibrium was achieved between the photocatalyst and MO. This is followed by illumination either under solar simulator or under natural sun light. The degradation of MO was studied by measuring the concentration of MO with a UV–vis spectrophotometer at regular intervals of time. The UV–vis spectra are shown in Figure 9. The corresponding kinetic curves are shown in Figure 10, which indicates that in all the cases the reaction follows zero-order kinetics similar to other reports in literature [60].

The degradation efficiency of both the photocatalysts was evaluated on the basis of initial and final concentration of the dye by monitoring the main absorption peak (λ = 454 nm) of MO. So degradation rate of the MO can be calculated by applying following equation [61]:


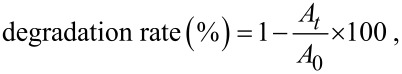


where *A_t_* and A_0_ are the absorbance at reaction time *t* and *t* = 0, respectively.

**Figure 11:**
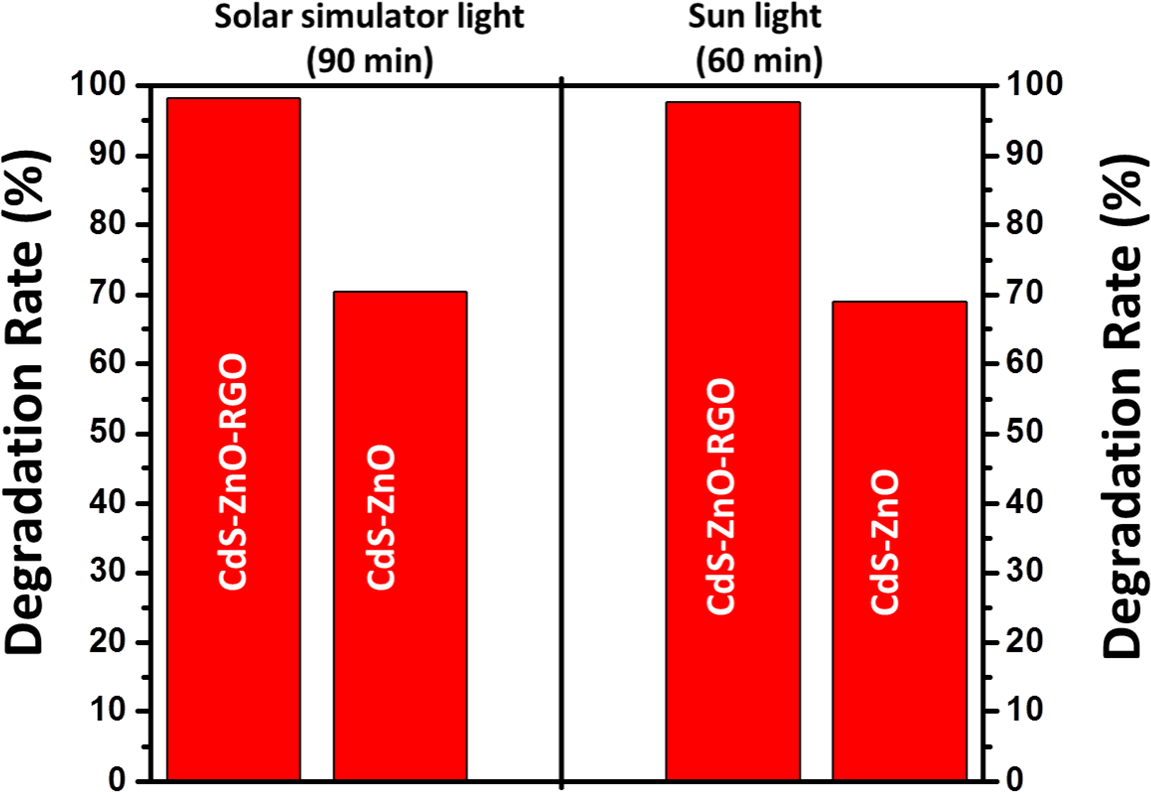
Histogram showing the degradation rate (%) of MO under visible light irradiation from a solar simulator and natural sun light illumination.

In accordance with the above equation, with CdS–ZnO–RGO ternary photocatalyst about 98% of dye was degraded in 90 min, under visible light illumination from a solar simulator, but with CdS–ZnO binary photocatalyst, only about 70% of dye was degraded. The degradation of the same concentration of MO was also investigated under natural sunlight illumination having an intensity of 9.5 × 10^4^ lux. Substantial improvement in the photocatalytic activity can be observed for both binary and ternary nanocomposites as shown by the histogram in Figure 11. It is noteworthy that about 98% degradation of the dye could be achieved within 60 min, when the CdS–ZnO–RGO ternary nanocomposite was used as the photocatalyst, which is 30 min shorter than under the simulated solar light. Similarly, within 60 min the CdS–ZnO binary nanocomposite degraded about 70% of the dye, while it took about 90 min under the visible light irradiation from a solar simulator. The enhanced performance of the nanocomposites can be attributed to the fact that the natural sun light has both UV and visible light components in it, so both of the semiconductor materials (CdS NP and ZnO NR) are active and electron–hole pair formation occurs in both. Hence, the generation of higher number of electron–hole pairs, their effective charge separation and charge transfer are the major factors responsible for the better activity of the photocatalysts under natural sunlight compared to the visible light irradiation using a solar simulator.

### Mechanism of photocatalytic activity

**Scheme 1:**
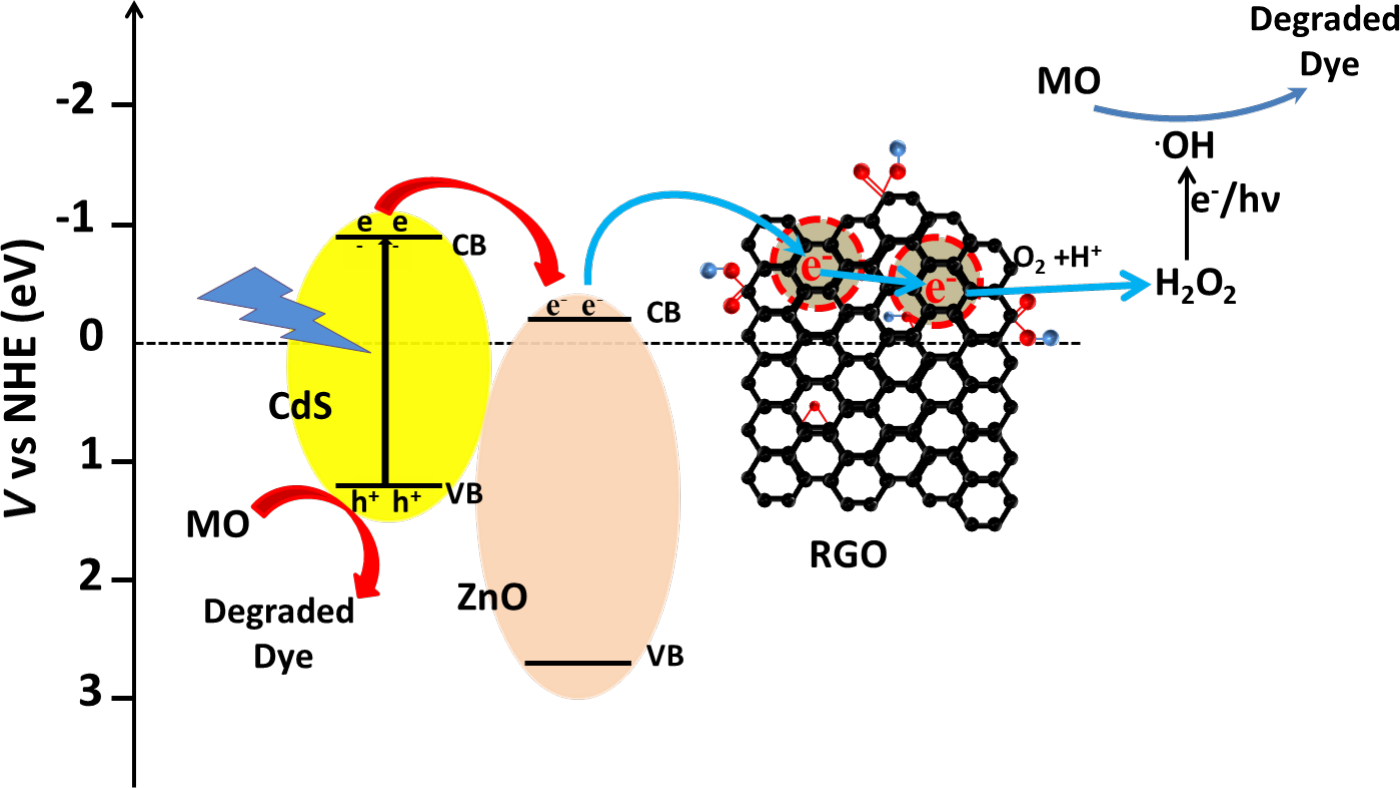
Possible mechanism of the photocatalytic activity of CdS–ZnO–RGO ternary nanocomposite for degradation of MO under visible light irradiation from a solar simulator.

**Scheme 2:**
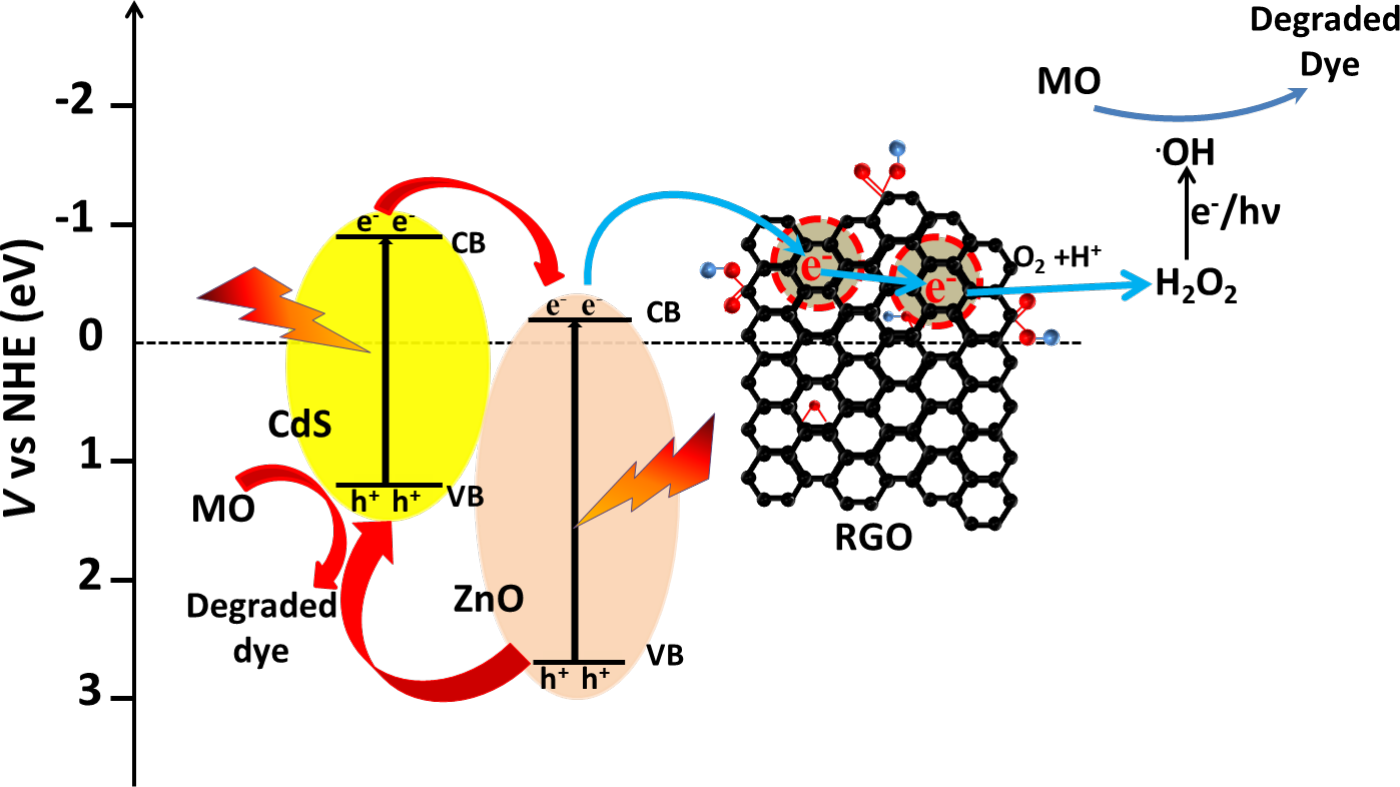
Possible mechanism of the photocatalytic activity of CdS–ZnO–RGO ternary nanocomposite for degradation of MO under irradiation from natural sun light.

The possible mechanisms of the photocatalytic activity of CdS–ZnO–RGO ternary nanocomposite for degradation of MO under visible light irradiation from a solar simulator and natural sun light are illustrated pictorially in Scheme 1 and Scheme 2, respectively.

Under visible light illumination, electron–hole pairs are generated in conduction band (CB) and valence band (VB) of CdS. The CB potential of CdS are *E*_CB_ = −0.66 eV vs NHE, which is more negative than the *E*_CB_ of ZnO [62–63]. This relative position of CB of CdS with respect to ZnO leads to the charge transfer from CB of CdS to the CB of ZnO [22]. The charge transfer dynamics in the CdS–ZnO composite material have been investigated before and it has been reported that this transfer occurs very rapidly in less than 18 ps [64]. The work function of ZnO is 5.2–5.3 eV and that of graphene is 4.5 eV [65]. Thus electrons that are transferred to the CB of ZnO are rapidly transferred to the RGO, as the Fermi level of graphene (−0.08 V vs NHE) is more positive than the redox potential of O_2_/O_2_^−^ (−0.13 V vs NHE) but more negative than the redox potential of O_2_/H_2_O_2_ (+0.695 V vs NHE) [66]. This demonstrates that electrons from graphene can react with O_2_ and H^+^ ions to produce H_2_O_2,_ which further decomposes in the presence of light to generate hydroxyl radicals (**^•^**OH) [67]. Also ZnO is inactive under visible light, so hole formation takes place only in the VB of CdS. As the VB edge of CdS is more negative than the standard redox potential of ^•^OH/OH^−^ (2.38 eV vs NHE) and ^•^OH/H_2_O (2.72 eV vs NHE), ^•^OH cannot be generated by reacting with H_2_O molecules. The **^•^**OH radicals are responsible for the dye degradation into CO_2_ and H_2_O [68–69]. The enhanced photocatalytic activity of the RGO-supported CdS–ZnO nanocomposite could be attributed to the presence of RGO, which has a very good dye adsorption and fast electron transport ability [70–71]. The enhanced activity under natural sun light could be attributed to the combined activity of both CdS and ZnO, wherein CdS absorbs in the visible region and ZnO absorbs in the UV region, and charge carriers are generated in both of these semiconductors. In this case, in addition to the electron transfer from the CB of CdS to the CB of ZnO, simultaneous hole transfers also occur from the VB of ZnO to VB of CdS, as the VB of CdS is more cathodic than the VB of ZnO [72]. Thus the recombination of photogenerated charges is suppressed more effectively under natural sunlight illumination, where both the semiconductor materials are active and more electron–hole pairs are generated, and their effective separation and rapid transport to the reaction site are responsible for the enhanced activity of the photocatalysts.

Overall, the improved photocatalytic activity of the ternary nanocomposites could be mainly attributed to the better adsorption capacity of graphene, rapid charge transfer at the semiconductor interface and then to graphene, which degrade the adsorbed dye on its surface. This whole proposed mechanism can be formulated as [67,73–74],


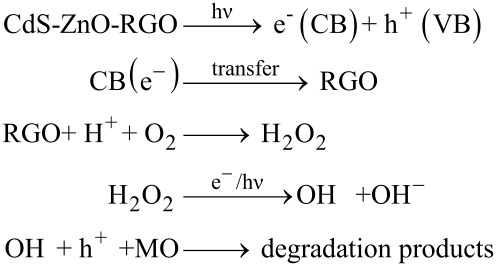


Furthermore, Table 1 presents the comparison of the photocatalytic activities of CdS–ZnO–RGO, CdS–RGO and ZnO–RGO nanocomposites prepared by different routes for the degradation of various pollutants, including MO. Table 1 also reveals that our reported CdS–ZnO–RGO nanocomposite exhibits an enhanced photocatalytic performance compared to other reports based on similar materials. The reason for better photocatalytic performance has been discussed in detail above in the mechanism section.

**Table 1:** Comparison of photocatalytic activities of CdS–ZnO–RGO based nanocomposites for degradation of pollutants, including methylene blue (MB), rhodamine B (Rh B) and MO.

photocatalyst	synthesis route	irradiation source	pollutant concentration	*t*_completion_ (min)	ref.

CdS–ZnO core-shell coupled with RGO	soft chemical route	simulated solar radiations	3.0 × 10^−5^ M (MB)	80	[75]
ZnO–RGO–CdS	hydrothermal	11 W UV lamp	1.0 × 10^−5^ M (MB)	ca. 240	[76]
ZnO–graphene	solvothermal	halogen lamp	6.0 × 10^−6^ M (MO)	90	[32]
3D grapene–ZnO NR	CVD and hydrothermal	UV light 300 W	5.0 × 10^−3^ M (MO)	ca. 60	[77]
graphene–ZnO NR film	hydrothermal	250 W Hg lamp (UV)	3.0 M (MB)	ca. 450	[78]
CdS–RGO composite	wet chemical method	UV	unknown (MO)	120	[79]
RGO–ZnO NR composite	hydrothermal	500 W Hg Lamp (UV)	1.0 × 10^−5^ M (Rh B)	90	[37]
CdS–graphene composite	hydrothermal	500 W Xe lamp	3.0 × 10^−5^ (MO)	360	[80]
CdS–ZnO–RGO	hydrothermal	visible light	1.0 × 10^−5^ M (MO)	90	this work
		sunlight	1.0 × 10^−5^ M (MO)	60	

## Conclusion

In this work, we have prepared and thoroughly characterized CdS–ZnO semiconductor nanostructures both with and without RGO support. Their photocatalytic activity towards the degradation of methyl orange dye, has been investigated both under visible light irradiation from a solar simulator and under natural sunlight. The obtained results show the significant role played by the RGO support and the source of irradiation on the photocatalytic activity of the mixed metal chalcogenide nanocomposites. The RGO-supported CdS–ZnO nanocomposites exhibits considerably better photocatalytic activity compared to its unsupported counterpart, which could be attributed to the enhanced photo-generated charge separation, facile charge transfer and strong adsorption of dye on to RGO. In addition, superior photocatalytic activity was observed for the nanocomposites irradiated under natural sunlight than visible light from solar simulator. This could be ascribed to the higher generation of electron–hole pairs, their effective separation and rapid transport to the reaction site. In this case, both the semiconductors are active, in their respective wavelength domains, as sunlight is comprised of both UV and visible light regions. This work not only demonstrates the role of the RGO support and irradiation source on the activity of photocatalysts, but also paves way for tailoring the photocatalytic activity of semiconductor nanostructures in general.
